# The British Journals: Surgery

**Published:** 1839-07

**Authors:** 


### SURGERY.

### *Case of Laceration of the Perineum cured by Operation.* By Robt. Davidson, Esq.

[This case is extremely creditable to Mr. Davidson. It may admit of question whether the aperient medicines ought not to have been administered earlier.]

The subject of this case was a lady about twenty-nine years of age; the accident took place in her second accouchement. The laceration of the perineum was complete from the vulva to the anus, and extended from half to three quarters of an inch into the recto-vaginal septum; the consequence of this was an involuntary discharge of fæces, to the great distress of the patient and her friends. Feeling much anxiety about the case, from the knowledge of the want of success which had so frequently attended the operation, I referred to the cases recorded on the subject.

The able memoir by Baron Roux, read before the Académie Royale des Sciences, in January, 1834, fell under my notice, and as the result, in several cases, had proved so very satisfactory, I determined on adopting the plan he pursued,—that of the quilled suture,—with a little variation, which I will presently describe.

Having explained my views to Dr. Henry Davies, whom I had the pleasure to meet in consultation, he perfectly agreed with me on the method I proposed to adopt; and although only twelve days had elapsed since the accident, the patient was so anxious to have the operation performed, that I did not hesitate to do it, having had the bowels previously well opened by means of a purgative and enemas. On the 6th November, in company with Dr. Henry Davies, I performed the operation in the following manner: I passed deeply a strong double ligature, by means of a common curved needle, close by the edge of the rectum, and another rather more than half an inch apart from the first, towards the vagina, after which I pared the edges of the wound, which I had not previously done, that I might not be annoyed by the oozing of blood, so as to be enabled to place the ligatures more accurately. The ligatures being introduced, I employed, as cylinders, two pieces of elastic gum catheter, about an inch and a half in length, one of which was placed in the loops which the double ligatures formed on one side, and the other between their separate ends, tying them firmly upon the cylinders. Baron Roux found in his cases that the use of the quilled suture caused an eversion of the edges of the wound; to remedy this he had recourse to several small sutures, at different points, between the different ligatures. To effect the same object, and also with a view of keeping the divided parts more closely and firmly in contact, I adopted the following plan, the materials for which I had prepared previous to the operation. I armed a curved needle with a piece of narrow tape, four inches long, having a knot at one end; this was passed down each end of both cylinders about half an inch, and brought outwards, the tape being prevented slipping through by the knot; the tapes then were thus placed in such a position as to be intermediate to the ligatures; this being done I turned the cylinders gently towards the edge of the wound, and tied the corresponding tapes over it, which I think rendered it much more solid than any number of small ligatures could have done. After the completion of the operation there was so little tension of the parts that we thought it quite unnecessary to make the lateral incisions so strongly recommended by M. Dieffenbach. The wound was dressed with simple dressing, and a roller applied to the knees to guard against involuntary motion during sleep. The mistura cretæ, with the liquor opii sedativus, was given daily to restrain the action of the bowels, and severe diet ordered, to such an extent that during seventeen days nothing was allowed but gruel, and that in small quantities, with occasionally a little hard biscuit. This treatment, however, the lady endured with the greatest patience and fortitude.

The urine was drawn off night and morning, which, as she lay on her side, with the thighs flexed on the body, was frequently attended with no small difficulty.

No untoward symptom occurred, and on the seventh morning the ligatures were removed, when it was found that firm union had taken place along the whole extent of the wound, with the exception of a small fistulous opening near the edge of the sphincter, having no communication with the rectum, and producing no inconvenience to the patient. After nine or ten days the urine was passed without assistance, the parts being guarded by a pledget of linen; the small fistulous opening was touched night and morning with tinct. cantharidis, and occasionally with argenti nitras, with the best possible effect.

On the seventeenth day no evacuation from the bowels having taken place, we administered a purgative draught and laxative enemas, which producing no effect, were repeated; but so impacted had the fæcal matter become that there was much difficulty in accomplishing our object, so that we were obliged to have recourse to manual assistance. We were not without our fears as to the effect of the action of the bowels on the newly-united parts, but the union was so firm as to sustain no injury. When regular action was established, she was allowed gradually to move about, and at the end of six or seven weeks she resumed her usual habits. During the whole time the infant was applied to the breasts at intervals, sufficient to relieve them, and keep up the lacteal secretion, as she was anxious to continue nursing. *Lancet. May* 4, 1839.

---

*On the Power of the Periosteum to form new Bone.* By James Syme, Esq. Professor of Clinical Surgery in the University of Edinburgh.

The question (says Mr. Syme) which I propose to consider is—whether the periosteum, or membrane that covers the surface of bones, possesses the power of forming new osseous substance, independently of the bone itself? From the experiments which are related below, and from the observation of cases of necrosis, Mr. Syme thinks that there is evidence for putting beyond all question *the power of the periosteum to form new bone, independently of any assistance from the old bone.*

Exp. i. An inch and three quarters of the radius of a dog was removed, *with* the periosteum.

Exp. ii. An inch and three quarters of the radius of a dog was removed, *leaving* the periosteum.

The results of the two experiments were, that upon examination after the lapse of a considerable space of time, the bone was perfectly reformed where the periosteum *remained,* and where the latter had been *removed* the bone was not regenerated, but in its place was found a band of tough ligamentous texture.

Exp. iii. The periosteum was separated from the radius of a dog; a plate of metal was placed between the periosteum and bone; upon examination six weeks afterwards, a deposition of osseous substance was found *in* the periosteum, forming a bony plate exterior to the metal, and not connected with the old bone, excepting by means of the membrane.

Exp. iv. The periosteum was removed from the surface of the radius of a dog; the denuded bone was then covered with a layer of metal; upon examination six weeks afterwards, a tough capsular covering was found, inclosing the metallic plate, but not containing any osseous substance.

*Transactions of the Royal Society of Edinburgh.* Vol. xiv. Part 1. 1839.

[" It is quite an erroneous notion," says Müller,* "to imagine that one organized part may be the nutrient organ of another; for instance, that the substance of the bones is formed and nourished by the periosteum. *The osseous substance being itself organized, must itself assimilate the nutritive matters.* The idea that bones are formed by the periosteum, appears to me to be a barbarism unworthy the present state of physiology." We recommend to his notice the experiments of Mr. Syme.]

---

*Case of Dislocation of the Left Femur on the Pubes.* By John Dunn, Esq. Surgeon, Scarborough.

John Coverdale, aged forty-three, was employed on the 13th February, 1839, at a quarry, in Burniston, when about one ton of earth suddenly fell upon his body and right thigh, throwing him upon his back. A second fall of earth immediately succeeded to the first, but striking with great force upon the inside of the left thigh (the right being already fixed), dislodged the femur upon the pubes.

My pupil, Mr. Best, who first saw the patient, immediately discovered the nature of the accident; for there was a very distinct bony prominence on the pubes, a great depression in the usual situation of the great trochanter, the limb everted and separated from its fellow, and about an inch shorter in length. My partner, Mr. Travis, and myself, made arrangements for the patient to be brought to Scarborough, where more efficient attentions could be secured. On calling a consultation, which was obligingly attended to by Mr. Weddell and Mr. Wilson, we found that no proper apparatus for the reduction existed in the town; that the case was of such extreme rarity as never to have been seen by any of us, either in hospital or private practice, but the course to be pursued was laid down by Sir A. Cooper, in his work on Dislocations, and we felt no difficulty in our operations.

Having first placed our patient as upright as we could, on a firm bed, he was bled to 25 oz. As he had only been poorly fed, we did not feel warranted in further depletion. Tartar-emetic was then given in divided doses, to the amount of six grains, but neither syncope nor sickness supervened. He was now laid upon his right side, the left knee bent, and given in charge to an assistant, while a strong towel, passed within the groin to fix the pelvis, was attached to an iron bar, beyond and just before his head. For want of proper pullies, we had recourse to the rather unwieldy ones used in ships, having a single purchase with two single blocks. These were attached to a staple in the floor. A good deal of time was lost at first, in adjusting the apparatus to the knee, so as to give a sufficient firmness of attachment without acting too tightly and injuriously on the skin.

Extension was now made with considerable force, downwards and backwards, for about ten minutes, when the bandage at the knee gave way. Mr. Weddell then reapplied the cords very tight, and extension was renewed in the same direction. After persevering for about ten minutes or a quarter of an hour, without any apparent success, the head of the bone at length seemed to have moved. During this evident relaxation of the muscles, I directed the extension to be desisted from, and lifting up the thigh with a towel placed round it and the back of my neck, Mr. Weddell gently turned the knee inwards, making a lever of the limb. The head of the bone returned to its place, with a slight snap. The fever after the accident was scarcely perceptible. A little sickness and griping supervened, probably from the tartar-emetic; and an aperient was all that was required during the subsequent treatment. The patient was kept in bed three days, when he got up and walked across the room. I saw him about seven weeks after, when I found him walking very well, able to rotate one limb as well as the other, and only complaining of a little weakness in his knee. The time which elapsed, from the occurrence of the accident to the reduction, was about seven or eight hours.

*Medical Gazette. May* 18, 1839.

* Müller's Physiology, by Baly, page 383.

